# Combination Therapy in Diabetes Mellitus Patients Attending Outpatient Department in a Tertiary Care Centre: A Descriptive Cross-sectional Study

**DOI:** 10.31729/jnma.7642

**Published:** 2022-12-31

**Authors:** Naresh Karki, Kamal Kandel, Kyushu Shah, Pravin Prasad, Jeevan Khanal

**Affiliations:** 1 Department of Pharmacology, Lumbini Medical College and Teaching Hospital, Tansen, Palpa, Nepal; 2 Department of Pharmacology, Institute of Medicine, Maharajgunj, Kathmandu, Nepal; 3 Department of Internal Medicine, Lumbini Medical College and Teaching Hospital, Tansen, Palpa, Nepal

**Keywords:** *diabetes mellitus*, *drug combinations*, *outpatients*, *teaching hospital*

## Abstract

**Introduction:**

Assessing anti-diabetic drug use patterns in hospitals is an important activity which helps to promote the rational use of drugs and may suggest measures to change prescribing habits for the better. This study aimed to find the prevalence of combination therapy in diabetes mellitus patients attending the outpatient department of a tertiary care centre.

**Methods:**

A descriptive cross-sectional study was conducted among 201 diabetes mellitus patients in the internal medicine department from 2 March 2022 to 30 June 2022 for a duration of four months after approval from the Institutional Review Committee (Protocol No: IRC-LMC-01/R-022). Diabetic patients prescribed at least one anti-diabetic drug in prescription forms were included. Socio-demographic profiles, clinical characteristics and anti-diabetic drug use pattern-related data were collected. Convenience sampling was used. Point estimate and 95% Confidence Interval were calculated.

**Results:**

Among 201 patients, 134 (66.66%) (60.14-73.18, 95% Confidence Interval) patients were given combination therapy. The most common combination therapy was metformin 500 mg and sitagliptin 50 mg. A total of 324 anti-diabetic drugs were used. The average number of drugs prescribed per patient was 1.6±0.7. The number of anti-diabetic drugs prescribed by generic name and from the national essential drugs list was 74 (22.83%) and 188 (58.02%) respectively. Biguanides were used in 176 (87.56%) patients.

**Conclusions:**

These findings were similar to some other studies conducted in similar settings. In most patients, combination drug therapy was commonly used. Among combination therapy, two drug combinations were more prevalent.

## INTRODUCTION

Diabetes mellitus is a serious health issue worldwide.^1^ World Health Organization has found that approximately four hundred million people have diabetes mellitus throughout the world, particularly higher in low-and middle-income countries.^1^ In Nepal, the prevalence of pre-diabetes and type II diabetes was estimated to be 10% and 19.4% respectively.^2^

Diabetes mellitus should be treated properly with insulin and/or oral anti-diabetic drugs to reduce mortalities and to prevent/minimize diabetic complications like nephropathy, neuropathy, cardiac diseases and retinopathy.^3-5^ The irrational use of drugs can lead to an increase in morbidity, mortality and cost of treatment.^4-6^ Therefore, the assessment of anti-diabetic drug use patterns in hospitals is an important activity that helps to promote the rational use of drugs and may suggest measures to change prescribing habits for the better.^6-8^

This study aimed to find the prevalence of combination therapy in diabetes mellitus patients attending the outpatient department in a tertiary care centre.

## METHODS

A descriptive cross-sectional study was conducted in the Outpatient Department (OPD) of Internal Medicine of Lumbini Medical College and Teaching Hospital (LMCTH) for the duration of 4 months from 2 March 2022 to 30 June 2022. Ethical approval was obtained from the Institutional Review Committee (Protocol number: IRC-LMC-01/R-022) and the permission for data collection has been taken from Internal Medicine Department of LMCTH. The patients with a diagnosis of type 1 diabetes mellitus, type 2 diabetes mellitus, gestational diabetes mellitus and diabetes mellitus due to secondary causes irrespective of age and gender who visited Internal Medicine OPD during the study duration and the patients prescribed one or more anti-diabetic drugs in OPD prescription forms were included in this study. While, the patients with a diagnosis of diabetes mellitus in prescription forms, but were not prescribed any anti-diabetic drugs, were excluded from the study. The data were collected using a non-probability, convenience sampling technique from OPD prescription forms.

The sample size was calculated by using the following formula:


$$\begin{array}{ll} \text{n} & ={\text{Z}}^{2}\times \frac{\text{p}\times \text{q}}{{\text{e}}^{\text{2}}}\\ & =1.96^{2}\times \frac{0.09\times 0.91}{0.05^{2}}\\ & =126 \end{array}$$

Where,

n = minimum required sample size
Z = 1.96 at 95% Confidence Interval (CI)
p = prevalence of use of combination therapy in diabetes mellitus patients, 9%^9^
q = 1-p
e = margin of error, 5%

The collected data were entered into pre-designed proforma which included socio-demographic profiles of patients (age, gender, hospital number and domicile), clinical characteristics of patients (diagnosis, number of diagnosis, presence of comorbidities, type of diabetes mellitus) and information related to antidiabetic drug use pattern (number of drugs use, group of drugs use, name of the drug use, use of drugs from Anatomical Therapeutic Classification for diabetes mellitus, monotherapy/combination therapy, route of administration, number of drugs used in generic name, fixed-dose combination of drugs and number of drugs used from national essential drug list).

The groups of anti-diabetic drugs were sorted on the basis of Anatomical Therapeutic Classification (ATC); Main Group A: Alimentary Tract and Metabolism as A10AB (Fast acting insulin), A10AC (Intermediate acting insulin), A10AD (Intermediate or long acting + Fast acting insulin), A10AE (Long acting insulin), A10BA (Biguanides), A10BB (Sulfonylureas), A10BF (Alpha Glucosidase Inhibitor), A10BG (Thiazolidinediones), A10BH (Dipeptidyl Peptidase-4 Inhibitor) and A10BJ (Glucagon like Peptide-1 analogue).^10^

The National List of Essential Medicines Nepal 2021 (sixth revision) was reviewed for the assessment of the use of anti-diabetic drugs from national essential drug list.^11^ Both the main list and complementary list of national essential drugs list were taken into consideration. The average number of drugs used per patient was calculated by dividing the total number of drugs used by the total number of patients. The percentage of drugs prescribed in the generic name was calculated by dividing the number of drugs prescribed in the generic name by the total number of drugs used, multiplied by 100. Likewise, the percentage of drugs used from the essential drug list was calculated by dividing the number of drugs prescribed which were in the essential drug list by the total number of drugs used, multiplied by 100. All the research-related activities and information were kept confidential by using code numbers and hospital numbers for the identity of patients.

Data were entered and analysed using IBM SPSS version 18.0. Point estimate and 95% CI were calculated.

## RESULTS

Among 201 patients, 134 (66.67%) (60.15-73.19, 95% CI) patients were given combination therapy.

Among 134 patients, 71 (52.99%) patients were male and 106 (79.11%) patients were above 50 years of age. Similarly, 122 (91.04%) patients belonged to type II diabetes mellitus. In 117 (87.31%) patients, diabetes was associated with co-morbid diseases. Hypertension was the most common co-morbid disease presented in 86 (64.17%) patients. Moreover, 98 (73.13%) patients were prescribed in fixed-dose combination. Of which, metformin 500 mg+sitagliptin 50 mg was commonly used in 81 (60.44%) patients (Table 1).

**Table 1:** Socio-demographic profile and clinical characteristics of diabetic patients (n= 134).

Variables	n (%)
Age (in years)	
≤50	28 (20.90)
>50	106 (79.10)
Gender	
Female	63 (47.01)
Male	71 (52.99)
Domicile	
Rural	105 (78.36)
Urban	29 (21.64)
Presence of co-morbidities	
Yes	117 (87.31)
No	17 (12.69)
Type of diabetes mellitus	
Type I	12 (8.96)
Type II	122 (91.04)
Fixed dose combination	
Yes	98 (73.13)
No	36 (26.87)

**Figure 1:**
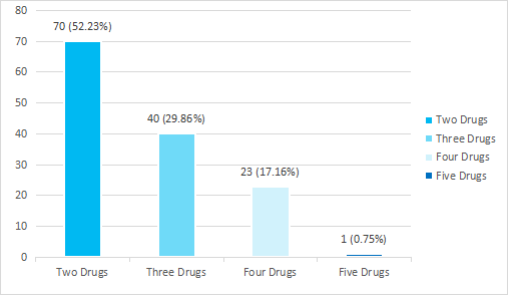
Pattern of use of antidiabetic drugs in combination therapy (n= 134).

The average number of drugs prescribed per patient was 1.6±0.7. Seventy (52.23%) of the patients were prescribed with two drugs combination therapy (Figure 1).

The number of anti-diabetic drugs prescribed in generic name was 74 (22.83%). While, 188 (58.02%) patients were prescribed anti-diabetic drugs listed in National List of Essential Medicines Nepal 2021 (sixth revision).

## DISCUSSION

This study demonstrated that two-third of the patients were prescribed combination therapy. Among combination therapy, two drugs combination was the most frequent. The majority of the diabetic patients were above 50 years of age and were having type II diabetes mellitus. These findings were corresponding with other studies.^12-15^ The asymptomatic presentation of type II diabetes could be the possible reason for diagnosis at old ages. The important risk factors for the development of type II diabetes are family history, sedentary lifestyle, stress and obesity. The present study supports the fact that diabetes tends to increase with increase age.

The current study also reported that the frequency of occurrence of diabetes was slightly higher among the male. The finding was comparable with the findings of some other studies.^16-18^ However, few studies determined to the finding that diabetes mellitus was predominant in female gender.^12-15^ Because males are more likely exposed to the risk factors of diabetes mellitus like obesity, stress, smoking and alcohol consumption which could be the possible reasons for prevalent of diabetes among them.^16^ Further, the study exhibited that hypertension was the most common co-morbid disease like showed in other studies.^17,18^ The patho-physiology behind the development hypertension in diabetes patients is the vascular remodeling with the progression of disease that lead to vascular resistance and increase in blood pressure.19 Beside this, increase in vascular fluid volume due to insulin-resistance associated hyperinsulinemia in type II diabetes is the another reason.^19^

Further, the study also showed that oral anti-diabetic drugs were used more frequently than injectable insulin because many patients were diagnosed as type II diabetes mellitus. Oral anti-diabetic drugs, exercise and dietary control are the main stays for the management of type II diabetes mellitus. Biguanides (metformins) were commonly prescribed in this study. Other studies also demonstrated similar findings.^4-6^ While, in a few studies drugs from the sulfonylurease group were used commonly.^15,18^ Metformin, which commonly works by inhibiting hepatic glucose production in the human body, is the first choice of drug for doctors to treat type II diabetes mellitus because of its advantages over other oral anti-diabetic drugs. Unlike sulfonylurease, metformin reduces elevated blood glucose levels without producing hypoglycemia and can be used in obese diabetic patients also.

In this study, nearly two third of the patients were prescribed combination drug therapy for diabetes mellitus which was comparable with the findings of some other studies.^5,7,12,13,16^ Similar Satpathy and Jimoh et al showed that two drugs combination was used often which was similar to the result of our study.^5,13^ However, in a few other studies only a single drug was used.^17,18^ Here, combination drug therapy means two or more drugs in different tablets as well as two or more drugs in the same tablet preparation which is also called a fixed-dose drug combination. Generally, two or more drugs are used in diabetes mellitus when the single drug is not able to achieve the recommended target level of plasma glucose. The rationale behind the use of combination therapy is also to produce synergistic actions with different mechanisms of actions of drugs and to reduce the doses of drugs which help to minimize adverse effects and cost of the treatment. Similarly, combination drug therapy in single tablets also strengthens the adherence of drugs in old age.

Furthermore, the study also found that the brand names of drugs were used by the majority of patients. This finding was also found in a few other studies.^5,6,16^ Contrary, the other two studies showed that generic prescriptions were commonly used.^4,17^ The practice of prescribing drugs by generic name is very much useful practice in hospitals because this helps to reduce the cost of drugs, improve access to essential drugs prescription and enables better communication between doctors, paramedics, nurses and pharmacists. Yet, the brand name of medicines is more popular among healthcare providers because of the influence of drug manufacturing companies. Likewise, the present study also demonstrated that half numbers of the total drugs used were listed in the National List of Essential Medicines Nepal 2021 (sixth revision). This finding is corresponding with a few other studies.^17,18^ The practice of using medicines from the essential drug list points out better therapeutic response, minimum adverse drug reactions and maximum cost-effectiveness of the drugs. Also, essential medicines satisfy the healthcare needs of the entire population nationwide as well as worldwide. Usually, the drugs used in the generic name and from the national essential drug list are the important components of rational use of medicines.

The researchers did not follow up with the patients to assess the actual drug use by the patients and adherence of patients to the drugs. This is one of the drawbacks of the study. Also, the single hospital-centred, short duration and convenience sampling technique are other limitations which restrict the generalization of the findings. However, the researchers expect that this study has made small basis to conduct similar studies in a larger population and multiple centres with follow-ups in the future.

## CONCLUSIONS

The prevalence of the use of combination therapy in our study was comparable with the findings of some other studies conducted in similar international settings. The study also summarizes that the two drug combinations were commonly accounted for in the treatment of diabetes mellitus. Biguanide (metformin) was the most commonly used along with another group of oral antidiabetic drugs. Besides these, the majority of the drugs were prescribed in generic names and from the national essential drug list of Nepal which are the necessary components of rationale use of medicines.
